# The evolution of zero-sum and positive-sum worldviews

**DOI:** 10.1073/pnas.2504339122

**Published:** 2025-08-05

**Authors:** Sergey Gavrilets, Paul Seabright

**Affiliations:** ^a^Department of Ecology and Evolutionary Biology, University of Tennessee, Knoxville, TN 37996; ^b^Department of Mathematics, University of Tennessee, Knoxville, TN 37996; ^c^Institute for Advanced Study in Toulouse, Toulouse School of Economics, Université Toulouse Capitole, Toulouse 31000, France; ^d^Department of Social and Behavioral Sciences, Toulouse School of Economics, Université Toulouse Capitole, Toulouse 31000, France

**Keywords:** evolution of worldviews, cooperation, competition, cultural authorities, cultural tightness

## Abstract

People and cultures differ in the extent to which they view the world as a zero-sum environment (where one person’s gain is another’s loss) or a positive-sum environment (where certain actions can benefit everyone). These beliefs shape individuals’ willingness to work, invest, collaborate, or show hostility toward out-groups, and accept or reject various social policies. We model dyadic interactions in a heterogeneous population where individuals biased toward a zero-sum worldview are more likely to invest in competition, while those biased toward a positive-sum worldview are more likely to invest in cooperation. The environment alternates stochastically between cooperative and competitive states. Without social influence, the more accurate worldview yields higher utilities and spreads throughout the population. However, assortative matching by bias can favor the positive-sum worldview even if a positive-sum environment is somewhat less likely. With peer conformity, inaccurate worldviews can persist after a structural change in the environment, leading to cultural evolutionary mismatch. In the presence of cultural authorities who can alter beliefs, either both worldviews can coexist or one excludes the other. Moreover, when assortative matching and conformity interact, authorities may profit by amplifying individuals’ biases, creating enclaves of similarly biased people who can pay the authorities enough to make investment in persuasive technology economically viable. Cultural evolutionary mismatch is more likely in cultures marked by strong peer conformity and high responsiveness to authority when the authority promotes a suboptimal worldview. This study demonstrates how real-world conditions, peer influence, and authority interventions can perpetuate or shift zero-sum and positive-sum worldviews-at times leading to inaccurate beliefs.

## Significance

Beliefs about whether the world is a zero-sum or a positive-sum environment vary across individuals and cultures, and affect people’s willingness to work, invest, and cooperate with others. We model interaction between individuals who are biased toward believing the environment is zero-sum, and those biased toward believing it is positive-sum. Beliefs spread through natural and cultural selection if they lead individuals to have higher utilities. If individuals are matched randomly, selection leads to the more accurate beliefs driving out the less accurate. Nonrandom matching and conformity biases can favor the survival of inaccurate beliefs. Cultural authorities can profit from creating enclaves of like-minded individuals whose higher bias drives out the more accurate beliefs of others.

From geopolitical conflicts to workplace dynamics, the perception of interactions as zero-sum or positive-sum fundamentally shapes human behavior. Belief in a zero-sum world is a phenomenon where people are predisposed to think that any improvements in their situation in life can come only at the expense of others, and conversely that gains to others must be at their own expense. It can be contrasted with belief in a positive-sum world, sometimes referred to as “win-win,” in which people are predisposed to think that gains to themselves can come at the same time as gains to others, and may even be facilitated by gains to others. Across domains as diverse as war and terrorism, trade and migration, climate change, and employment, parties’ worldviews shape their negotiation positions and can significantly influence outcomes.

It has been proposed that zero-sum thinking can be understood as “a generalized belief about how the world works” that “predisposes one toward zero-sum thinking, and its cognitive and strategic consequences, across situations and domains” (1). This worldview has been associated with the so-called Dark Triad of personality traits (psychopathy, machiavellianism, and narcissism) (2, 3). It is therefore likely to discourage people from trusting others (4–7) and from productive effort on their own behalf, because they fear the envy of others (8). Individuals with this worldview often show a diminished willingness to share or contribute to public goods (9, 10). It reinforces in-group favoritism and out-group hostility, as out-groups (such as immigrants) are frequently perceived as threats to limited resources (11). It can lead to conflicts in the implementation of social policies, as in the United States where reductions in bias against Black people have been perceived by many Whites (but not by Blacks) as implying necessarily an increase in bias against Whites (12).

These research findings imply that a predisposition toward perceiving the world as positive-sum encourages collaboration, with people more willing to engage in cooperative ventures and contribute to public goods, knowing that collective efforts can expand resources and create shared gains. It will also reduce in-group bias and out-group hostility, promoting inclusivity and trust across diverse groups. Recognizing the potential for win-win outcomes has been associated with a “growth mindset” (13), leading to innovation, more sustainable relationships, and the ability to address complex challenges collaboratively.

Countries and cultures differ significantly in the incidence of these worldviews (9). Religious movements have at different times and places laid very different emphasis on the positive-sum or zero-sum perspectives in the messages they send to their members (14). Both the Bible and the Quran contain verses emphasizing that believers should have an open, trusting attitude to life (“Take no thought for the morrow,” “whoever is mindful of Allah, he will make a way out for them”). Both also emphasize the enemies that lurk in wait for believers (“Your adversary the devil, as a roaring lion, walketh about, seeking whom he may devour,” “We made for every prophet an enemy among the criminals”).

The differences in worldviews may arise in response to the objective state of the environment—including aggregate resource availability (scarcity or abundance), and the extent of competition for these resources between socioeconomic, ethnic, political, religious, or territorially defined groups. For example, Chinoi et al. (14) use a survey of 20,400 US residents to show that “Zero-sum thinking can be traced back to the experiences of both the individual and their ancestors, encompassing factors such as the degree of intergenerational upward mobility they experienced, whether they immigrated to the United States or lived in a location with more immigrants, and whether they were enslaved or lived in a location with more enslavement” (p.1). Worldviews may also be a product of social influence—the interactions of individuals with others, the degree to which they associate in enclaves of the like-minded, the strength of pressures for conformity, the prevalence of cultural norms and societal narratives, and the reach of cultural authorities (15–17). A third set of factors includes individual personality traits, as well as diverse prior life experiences (such as economic hardship or social marginalization) that may affect current cognitive biases (18, 19). These factors interact dynamically to influence whether people view interactions as competitive or cooperative, fixed- or positive-sum (10).

We are interested in the extent to which beliefs align with the realities of the environment under the influence of natural selection (20, 21). It seems unlikely that there is an unconditional “power of positive thinking,” to cite the title of a famous self-help book published in 1952 (22). Believing in a positive-sum world might be a mistake if it leads people to behave in ways that are not appropriate to a zero-sum environment, and vice versa.

However, social influence might steer the evolution of worldviews away from the most accurate representation of the environment. Assortative matching—preferential interactions with individuals sharing similar beliefs or personality traits—can foster cooperation within like-minded groups (23, 24), but it could also perpetuate false or biased beliefs by creating echo chambers. Social norms, conformity, and the tug of social identity frequently act to align individual behaviors and beliefs with those of the majority (17, 25). They may also create polarization (26). In either case, they may thereby perpetuate outdated beliefs that no longer reflect current realities, resulting in reduced payoffs following environmental changes. This phenomenon, known as cultural evolutionary mismatch, highlights how entrenched norms can become maladaptive over time (27–29).

Cultural authorities often exert great effort to persuade others to change their beliefs about the world’s payoff environment. Those who speak in support of the positive-sum mindset include entrepreneurs trying to persuade the employees of a business to adopt a growth mindset (30), and political and cultural leaders trying to convince different groups in society that “we are all in this together” (31, 32). Those who argue for a zero-sum mindset include those who say of immigrants that “They’re taking our jobs. They’re taking our manufacturing jobs” (33).

Do such efforts at persuasion work? Media organizations might passively reflect rather than actively shape the attitudes of those who consume their content. Measures of the “slant” of news outlets show that readers gravitate to outlets whose slant reflects their views, rather than the slant forming those views (34). When news content on Italian television shifted to the political right after the 2001 election of Silvio Berlusconi, “viewers responded to these changes by modifying their choice of favorite news programs....while those viewers who continued to watch public channels were eventually exposed to a more right-leaning news coverage, this effect is offset in part by an opposite effect on those viewers who switched channels and ended up being exposed to a more left-leaning coverage” (35).

However, the fact that viewer response can potentially offset the impact of partisan news does not imply that partisan news has no effect at all. The staggered roll-out of cable channels in the United States has been used to estimate that the launch of Fox News, even allowing for selection into viewership by prior partisan leanings, resulted in a six percentage point increase in votes for the Republican Party in the elections of 2008 (36).

The influence of cultural authorities depends not just on their efforts but also on the overall cultural tightness or looseness of a society (37). Tight societies, characterized by strong norms and low tolerance for deviance, exhibit higher conformity to peers and a stronger responsiveness to authority messaging, in contrast to loose societies, which are more permissive and flexible in their social norms. Cultural tightness can impact various characteristics of the population including the effectiveness of cultural authorities in promoting “maladaptive” worldviews and the degree of cultural evolutionary mismatch.

There has been theoretical work on the evolution of biased beliefs outside the context of worldviews. For example, ref. 38 has suggested that overconfidence can be strategically useful to individuals in persuading others, a hypothesis that has received some experimental support (39). Additionally, formal models of reasoning under misspecified beliefs have also been developed—for example, by exploring what happens when agents update using Bayesian reasoning over a set of prior beliefs that excludes the true probability distribution (40, 41). In such cases, the system can be in an equilibrium in which everyone has a fully updated but incorrect belief, but the system may be vulnerable to invasion by other agents with a more accurate set of prior beliefs.

Although references to “zero-sum” or “positive-sum” worldviews are common in evolutionary game theory, they are typically interpretive rather than literal. Researchers often map selfish or cooperative strategies onto everyday language about beliefs without explicitly modeling the evolution of worldviews. A notable exception is Bergeron et al. (8) who develop a model of what they call *effort-suppressing* beliefs, which formalizes an argument due to the anthropologist George Foster (42) to the effect that in traditional societies peasant farmers have an “Image of the Limited Good” according to which benefits to some individuals can come only at the expense of others. Related phenomena such as belief in “the evil eye” (43) might have similar effects. In their model, an individual’s benefit increases with their own effort but decreases with their partner’s effort. The rate at which the partner’s effort reduces this benefit reflects the degree of zero-sumness in the environment. Individuals differ in the strength of the demotivating beliefs they hold, modeled by a parameter that discounts the return to their effort. Bergeron et al. (8) show that if individuals assort based on these beliefs, the accurate (nondemotivating) belief goes extinct, while a specific type of demotivating belief becomes established in the population, despite being an inaccurate representation of the world. This occurs because, when the environment is partly zero-sum, high effort by one person often hurts their partner. Individuals with moderate “demotivating” beliefs exert intermediate effort. If people tend to interact with others who hold similar beliefs, then moderate-effort types are more likely to face others who also hold back. This allows them to avoid the fitness costs that high-effort types incur through wasteful competition, while still earning higher benefits than low-effort types who contribute very little. As a result, they outperform both extremes.

Here, we employ a simple mathematical model to examine theoretically the effects and interactions of key factors—environmental characteristics, assortative matching, conformity, and cultural authorities—on the population-level dynamics of competing worldviews that may be biased toward believing the world to be zero-sum or positive-sum. By a “biased” worldview we do not mean one that always believes the world to be of a particular kind, just one that believes this with a higher probability than is warranted by the facts.

To focus on these dynamics, we exclude between-individual differences, except for variations in these worldviews. While the average effects of individual idiosyncrasies can often be predicted (for example, individuals who have previously lost out in competition with others may develop a bias toward a zero-sum worldview), their impact on population-level outcomes is far more complex. Predicting such effects would require detailed knowledge of the distribution of individual traits, which is often impractical or infeasible to obtain.

Our model differs from that of Bergeron et al. (8) by considering a stochastic environment that alternates between zero-sum and positive-sum states, and by incorporating competitive and cooperative behaviors, peer conformity, and the influence of cultural authorities. In their framework the payoff environment is to some degree zero-sum and to some degree independent (that is, payoffs to individuals are independent of the actions of others). It is never cooperative. This helps them to explain why in some societies individuals underinvest in their own prosperity. It does not help to explain why individuals might behave more or less cooperatively. Yet it has been a persistent theme of economic writing going back to (at least) the 18th century that when individuals believe that the environment rewards cooperative behavior they will be more cooperative (44, 45).

The question in our paper is whether a plausible selection mechanism for worldviews will tend to favor convergence to the true degree of non-zero-sumness in the environment, or whether equilibria exist in which false worldviews can be supported in the long run by the behavior patterns they induce. To the assortment considered by Bergeron et al., we also add social influence and the biased messaging of cultural authorities. The interactions among these different forces lead to a rich set of equilibria, in some of which the error-minimizing worldviews can be challenged or even completely displaced by other, more biased worldviews.

## 1. Model and Results

### 1.1. Basic Model.

We consider a population of individuals living in a random environment that can be in one of two possible states: zero-sum, where increased payoffs to one player can only come at the expense of another (e=0), and positive-sum (e=1), where some configurations of strategies (“cooperation”) can lead to higher aggregate payoffs than others. The probability that the environment is in the zero-sum state at any given time is *q*, while the probability of being in the positive-sum state is 1−q.

Individuals are identical in all aspects except their worldviews, denoted by *θ*. There are two possible types of worldview. Individuals with θ=0 are biased toward perceiving social interactions as zero-sum. Individuals with θ=1 are biased toward perceiving social interactions as positive-sum. Their frequencies are *p* and 1−p, respectively, and these frequencies may evolve over time.

Agents’ bias toward a certain state shows itself in that, while always recognizing that state when it occurs, they sometimes believe it occurs when it does not. Specifically an agent’s bias in evaluating the environment’s state is measured by the parameter δ≥0, in the following way:

- “Competitively biased” agents of type 0 have the true belief that e=0 with probability 1, if e=0. If e=1, they have the true belief that e=1 only with probability 1−δ.
- “Cooperatively biased” agents of type 1, have the true belief that e=1 with probability 1, if e=1. If e=0, they have the true belief that e=0 only with probability 1−δ.

We assume that agents make independent errors. Agents interact in randomly formed pairs. Each agent can invest in competition (effort *x*) or cooperation (effort *y*). The payoff in environment *e* of an individual *i* interacting with an individual *j* is

[1] $$\begin{array}{c} \pi_{e}\left(i\right)=-\underset{\text{cost}}{\underbrace{\frac{1}{2}c_{0}x_{i}^{2}-\frac{1}{2}c_{1}y_{i}^{2}}}+\left\{\begin{array}{c} \underset{\text{benefit of competition}}{\underbrace{b_{0}\left(x_{i}-x_{j}\right),}}\;\text{if}\;e=0,\\ \underset{\text{benefit of cooperation}}{\underbrace{b_{1}\left(y_{i}+y_{j}\right),}}\;\text{if}\;e=1, \end{array}\right. \end{array}$$

where $b_{i}$ and $c_{i}$ are the benefit and cost parameters. That is, in the competitive environment, the agent with the larger (smaller) effort $x_{i}$ has a positive (negative) benefit, while in the cooperative environment, the agents play a linear public goods game. In both cases, the costs are quadratic in efforts so that they are small for small efforts but then rise rapidly. We do not impose a fixed total effort budget because the quadratic costs endogenously limit total effort, and our results depend only on relative payoffs, not on the specific mechanism that constrains effort.

In Eq. **1**, each player’s payoff is dependent on the other player’s action, but the marginal return to investing in either cooperation or competition does not depend on what the other player does. This means that the players choose their actions based on their beliefs about the state of the world, but not on any beliefs about what the other player thinks to be the state of the world.

In our approach, actions adjust on a fast time-scale converging to the simple best-response given the beliefs about the environment, while world-view biases update slowly via social learning (as in refs. 20, and 46, 47, 48, 49, 50). This mirrors empirical evidence that while behavioral responses shift from one encounter to the next, beliefs such as the zero-sum mindset display high longitudinal stability (1) and cross-cultural consistency (9).

In *SI Appendix*, we identify the corresponding Nash equilibria for *x* and *y* as well as the resulting payoffs for situations when both players have the correct belief about the environmental state or when one of them has an incorrect belief. We also show (*SI Appendix*, Eq. **S3**) that the average difference in payoffs between the two types, that is $s=\pi_{0}-\pi_{1}$ is

[2] $$\begin{array}{c} s=\left(r_{0}+r_{1}\right)\delta \left(2q-1\right), \end{array}$$

where composite parameters $r_{i}=\frac{b_{i}^{2}}{2c_{i}}$ for i=1,2 measure the payoffs of player *i* in environment *i* when they have the correct belief about the environmental state while their opponent has an incorrect belief. This immediately indicates that the type making fewer errors enjoys higher payoffs. If the competitive environment is more frequent (q>0.5), this advantage belongs to the competitively biased type 0. Conversely, if the cooperative environment 1 is more frequent (q<0.5), the cooperatively biased type 1 has higher payoff.

### 1.2. Evolutionary Dynamics.

We can now use the replicator equation (51–54) to describe the dynamics of the type frequencies over time:

[3] $$\begin{array}{c} \frac{\mathrm{dp}}{\mathrm{dt}}=sp\left(1-p\right), \end{array}$$

This equation emerges if individuals with higher payoffs leave more offspring who inherit their type or if individuals with higher payoffs are more often copied by young individuals. The former case describes natural selection, while the latter case corresponds to payoff-biased imitation. From Eq. **3** with parameter *s* defined as in Eq. **2**, we immediately conclude that the type making more errors (which will be type 0 if q<0.5 and type 1 if q>0.5) will eventually be eliminated from the population. In this case, there will be survival of the more accurate worldviews. The absolute value of *s* controls the rate of evolution. Larger payoff benefits relative to costs ($r_{i}$), higher error rates (*δ*), and greater imbalance in the frequencies of the two environments (|2q−1|) all contribute to increasing the rate of evolution.

### 1.3. Nonequal Error Rates.

Similar conclusions emerge if the two types differ in their likelihoods of errors, $\delta_{0}$ and $\delta_{1}$. This seems very plausible in real-life contexts, since the reasons why someone might overestimate the probability that the environment is zero-sum bear little relation to the reasons why someone might overestimate the probability that the environment is positive-sum. For example, the media might report cases of robbery with greater probability than they would report acts of kindness to strangers. Corporate fraud might attract greater attention than corporate generosity. Or, in a different setting, charities and churches might advertise their benevolence widely while doing their best to cover up cases of abuse. Some politicians might stress that immigrants are taking native workers’ jobs while underplaying the fact that they are also looking after those same workers’ elderly relatives. Entrepreneurs could be so focused on beating the competition that they overlook the extent to which they depend on their clients and suppliers. Children who have suffered abuse from their parents might overestimate the risks of abuse in society at large, or children who have been well treated by their parents might underestimate the risks posed by other adults.

With nonequal error rates, we show (*SI Appendix*, Eq. **S5**) that the expression for parameter *s* takes the form

[4] $$\begin{array}{c} s=\left(r_{0}+r_{1}\right)\left(q\delta_{1}-\left(1-q\right)\delta_{0}\right). \end{array}$$

The values $q\delta_{1}$ and $\left(1-q\right)\delta_{0}$ represent the overall error rates for type 1 and type 0, respectively, accounting for the frequencies of the different environmental states: Higher error rates matter less if they happen in states that occur less frequently. The model then predicts that the type with the lower overall error rate will spread in the population, while the type with the higher overall error rate will disappear from the population.

### 1.4. Assortative Matching.

Using a standard approach for modeling assortment (55), assume that a fraction *ρ* of the population matches with its own type and fraction 1−ρ matches at random with another member of the population. Parameter 0≤ρ≤1 measures the degree of assortment. Returning to the symmetric case of $\delta_{0}=\delta_{1}=\delta$, parameter *s* becomes (*SI Appendix*, Eq. **S7**):

[5a] $$\begin{array}{c} s=\underset{\mathrm{natural}\;\mathrm{selection}}{\underbrace{\left(r_{0}+r_{1}\right)\delta \left(2q-1\right)}}-\underset{\text{assortment}}{\underbrace{2\delta \rho (r_{0}q+r_{1}\left(1-q\right))}}. \end{array}$$

This implies that the maximum value of *q* ensuring that cooperative type 1 wins the competition changes from 1/2 to

[5b] $$\begin{array}{c} q^{*}=\frac{1}{2}\left(1+\frac{\rho }{1+\frac{r_{1}-r_{0}}{r_{0}+r_{1}}\rho }\right). \end{array}$$

The ratio in the last equation is always positive, which implies that $q^{*}>0.5$. Therefore, the condition for the cooperative type 1 to spread becomes easier to satisfy. With sufficiently strong assortative matching, the cooperative type can prevail even if the cooperative environment is less frequent than the competitive environment. This conclusion is intuitive, as we know from evolutionary biology that genetic relatedness—analogous to the degree of assortative matching in our model—promotes cooperation (56). However, the mechanism here is not the spread of preferences for cooperation, but rather the spread of worldviews—individuals who are biased toward believing the environment is more likely to be positive-sum than it actually is will tend to prosper because they are more likely to be matched with other individuals who, sharing the bias, invest more in public goods.

Notice that if type 0 is to eliminate type 1 (which occurs when s>0), a larger assortment *ρ* decreases the rate of evolution *s*. Conversely, if type 1 is to eliminate type 0 (which occurs when s<0), a larger assortment *ρ* increases the rate of evolution *s*.

It should finally be noted that not all matching mechanisms will tend to favor assortative matching. In some contexts individuals who believe the environment is zero-sum may strongly prefer to match with those who believe the opposite (since the latter will underinvest in competition). They may not be able to bring about this outcome (indeed it is far from clear what the equilibrium degree of assortativity or counterassortativity will be in such a situation), but its possibility should not be ignored.

### 1.5. Conformity With Peers.

So far, we have assumed that material payoffs were the only factor affecting the evolutionary dynamics of the population. However, humans also experience various psychological benefits and costs as a result of their actions. One of the most powerful forces affecting human decision-making is conformity, which leads to psychological benefits when an individual’s actions align with those of others, and to psychological costs when an individual’s actions misalign with those of others (57). Human predisposition to be influenced by the majority’s behavior or beliefs has evolved during our evolutionary past (58–60). Accordingly, we incorporate these psychological effects into the individual utility function, which in turn shapes the dynamics of worldview frequencies within the population. Notably, this differs from the assumption in the literature on indirect evolution (61, 62), where individual choices are based on subjective payoff functions, but the evolution of strategy frequencies is driven exclusively by material payoffs translated into differential fertility.

Specifically, we assume that besides payoffs specified by Eq. **1**, individuals receive additional utility proportional to the frequency of social partners sharing the same worldview. Given the degree of assortment *ρ*, we can write this utility as α[ρ+(1−ρ)p] for individuals choosing θ=0 and α[ρ+(1−ρ)(1−p)] for individuals choosing θ=1, where *α* is a constant nonnegative parameter measuring the strength of conformity. Then the difference in utilities related to conformity is α(1−ρ)(1−2p) which leads to a modified equation for *p*:

[6a] $$\begin{array}{c} \frac{\mathrm{dp}}{\mathrm{dt}}=\underset{\text{natural selection}}{\underbrace{sp(1-p)}}+\underset{\text{conformity}}{\underbrace{\alpha (1-\rho )p(1-p)(2p-1).}} \end{array}$$

In this setting, the assortment parameter *ρ* reduces the effect of conformity. With no assortment (i.e. if ρ=0), the conformity part of this equation is identical to the equation arising in the literature on imitation (63) or negative frequency-dependent selection and minority disadvantage in population genetics (64). Notice that higher assortment reduces the effect of conformity. Below we will denote by $\alpha_{e}$ the effective conformity rate, $\alpha_{e}=\alpha \left(1-\rho \right)$.

The addition of conformity to the model fundamentally changes its dynamics. In the models considered above, the population would evolve to the state with the highest payoff (at p=0 if s>0 or p=1 if s<0) independently of initial frequencies of the two types. With conformity, if it is strong enough, the state to which the population evolves depends on initial frequencies. Specifically, if

[6b] $$\begin{array}{c} \alpha_{e}>\left|s\right|, \end{array}$$

the population evolves to the state p=1 if the initial value of *p* is larger than $p^{*}=\frac{1}{2}\left(1-\frac{s}{\alpha_{e}}\right)$ and to p=0 otherwise. The critical value $p^{*}$ gets closer to 0.5 as $\alpha_{e}$ increases relative to the absolute value of *s*. If conformity is relatively weak ($\alpha_{e}<\left|s\right|$), then it merely delays the convergence to a higher-payoff equilibrium.

Similar conclusions arise if conformity is based not on the average worldviews in the population but instead on the average behavior (*SI Appendix*). However, in this case, the conformity term in Eq. **7** includes two additional parameters, *δ* and *q*, making the results less intuitive. Therefore, we focus on the former assumption below.

Without conformity, the basic model shows that asymptotically the agents will reach the error-minimizing worldview because that leads to the highest payoff. Natural selection works either via increased reproductive success of such agents or because they are imitated by others due to their higher payoff. If at some point environmental conditions change, the population will evolve and reach a new optimal worldview. However, in the presence of conformity the agents may get stuck with a non-error-minimizing worldview (and therefore with lower payoffs) after an environmental change. This would describe a “cultural evolutionary mismatch” (27–29). The degree of conformity is generally higher in tight cultures compared to loose cultures. Consequently, all else being equal, tight cultures are more prone to exhibiting a “cultural evolutionary mismatch.”

### 1.6. The Influence of Cultural Authorities.

Here, we consider the effect of a cultural authority interested in changing the culture of a population to be more cooperative or competitive.

We can think of cultural authorities as individuals in possession of a persuasive technology which allows them to “convert” agents of one type into another type at rate *β*. Then the equation for *p* changes to:

[7] $$\begin{array}{cc} \frac{\mathrm{dp}}{\mathrm{dt}} & =\underset{\text{natural selection}}{\underbrace{sp(1-p)\;}}+\underset{\text{conformity}}{\underbrace{\alpha_{e}p\left(1-p\right)\left(2p-1\right)}}\\ & \times \underset{\text{authority’s influence}}{\underbrace{\left\{\begin{array}{c} +\beta (1-p)\;\;\text{if worldview}\;\theta =0\;\;\text{is promoted},\\ -\beta p\;\;\text{if worldview}\;\theta =1\;\;\text{is promoted.} \end{array}\right.}} \end{array}$$

Notice that the overall effect of an authority is larger when the frequency of individuals being converted is higher.

For example, assume that worldview θ=0 is promoted. If there is no conformity (i.e., $\alpha_{e}=0$), Eq. **9** simplifies to:

$$\frac{\mathrm{dp}}{\mathrm{dt}}=sp\left(1-p\right)+\beta \left(1-p\right),$$

which is mathematically equivalent to the classical Bass model for the spread of innovations (65). The difference is that in the Bass model s>0 always, while in our case *s* can be positive or negative. If s>0, *p* asymptotically approaches 1, producing an S-shaped “adoption curve” similar to that in the Bass model. However, if s<0, *p* asymptotically evolves to β/|s|, which is smaller than 1 if |s|>β. This scenario occurs if the authority promotes a worldview that leads to lower payoffs. In this case, the population reaches a heterogeneous state where individuals with opposing worldviews coexist.

The coexistence of opposing worldviews is the most interesting outcome, which we consider next, returning to the general case where there is conformity as well as the presence of a cultural authority.

With s<0, so that selection favors the cooperative worldview θ=1, and the authority promotes the competitive worldview θ=0, the general Eq. **9** always has an equilibrium at p=1, where everybody rejects the error-minimizing worldview. This equilibrium is locally stable if

$$\alpha_{e}+\beta >\left|s\right|,$$

that is, if the joint effects of conformity with peers adjusted for assortment ($\alpha_{e}=\alpha \left(1-\rho \right)$) and the strength of messaging/propaganda (*β*) are stronger than the loss in payoffs |s|.

If the authority’s effort is strong enough, specifically if

$$\beta >\frac{(\alpha_{e}+{\left|s|\right)}^{2}}{8\alpha_{e}},$$

there are no other equilibria. Otherwise there are one or two additional heterogeneous equilibria with *p* between 0 and 1. If there is only one such equilibrium, it is stable for any initial conditions. If there are two such equilibria, the smaller one is locally stable together with the equilibrium at p=1, while the larger one is unstable and separates the domains of attraction of the two stable equilibria.

**Fig. 1.**
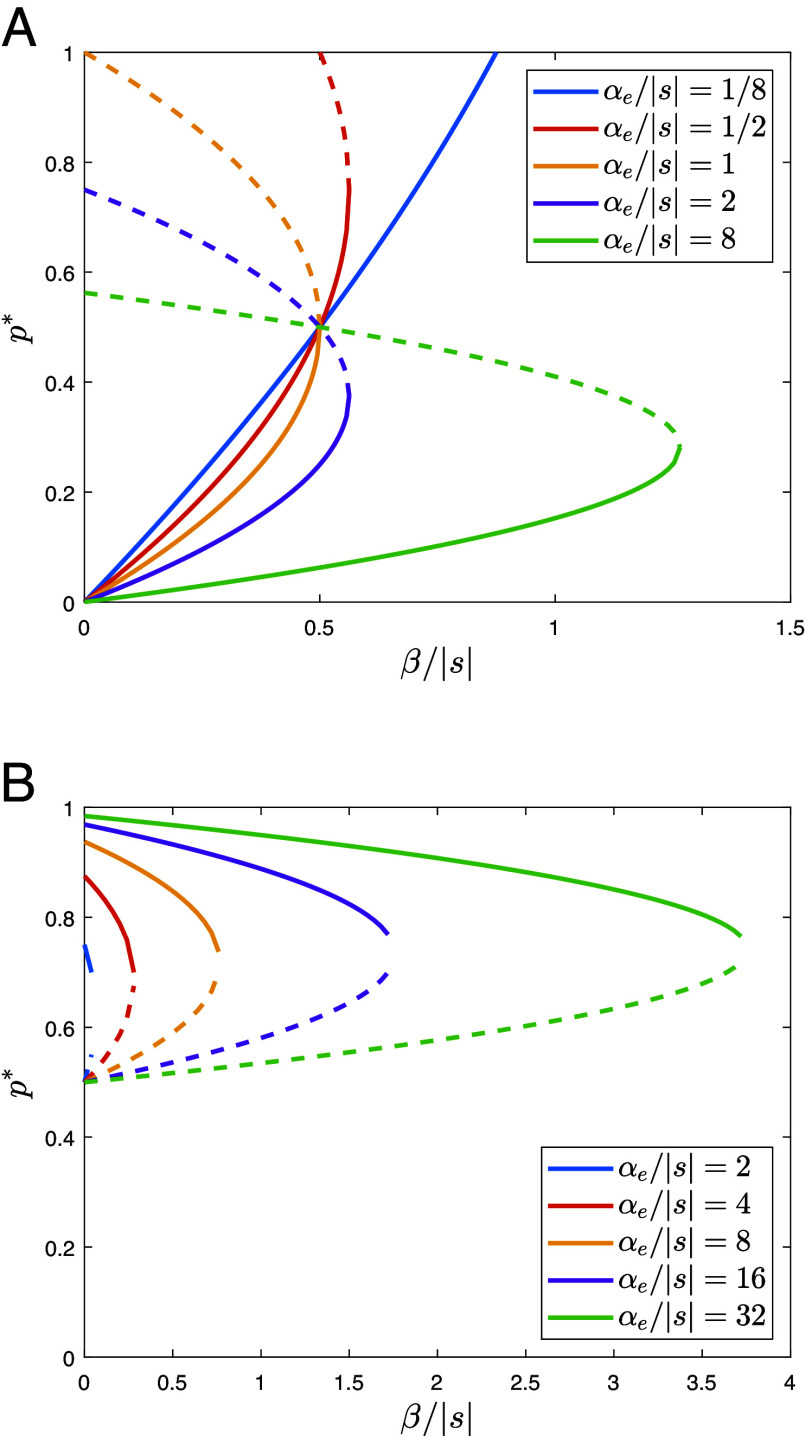
Effects of cultural authorities on the equilibrium frequency $p^{*}$ for different values of the scaled parameters β/|s| and $\alpha_{e}/\left|s\right|$, which measure the authority’s efficiency and the population’s conformity, respectively. (*A*) The authority promotes the zero-sum worldview. (*B*) The authority promotes the positive-sum worldview. In both cases, s<0, implying that the positive-sum worldview leads to higher material payoffs. Stable equilibria are represented by solid lines, while unstable equilibria are shown by dashed lines. Additionally, in (*A*), there is an equilibrium at p=1, where the positive-sum worldview disappears. This equilibrium is stable for values of β/|s| to the right of the intersection point between the corresponding solution branch and the horizontal line at p=1. In (*B*), there is an equilibrium at $p^{*}=0$, where the zero-sum worldview disappears. The heterogeneous equilibria can exist only for $\alpha_{e}/\left|s\right|>1$.

Fig. 1*A* illustrates the effects of model parameters on the existence and stability of heterogeneous equilibria. While we call *β* the “strength” of the authority’s effort, it is more intuitive to work with β/|s|, which we can call the “effectiveness” of that effort—it is the strength of the effort adjusted for the payoff disadvantage which that effort must overturn. The equilibrium frequency $p^{*}$ is increasing in the effectiveness of the authority’s effort. For small values of $\alpha_{e}/s$, there is a single heterogeneous equilibrium, and $p^{*}$ increases continuously with the propaganda effectiveness β/|s| until it reaches the value of 1. For larger values of α/s, there are two heterogeneous equilibria: one with smaller values of $p^{*}$, which is stable, and another with larger values of $p^{*}$, which is unstable. If the population initially resides at the stable equilibrium, increasing the propaganda effectiveness β/|s| beyond a certain threshold (at the intersection of the solid and dashed curves of the same color in Fig. 1) will cause a rapid transition to the equilibrium at p=1. This indicates that the system will exhibit tipping point dynamics.

In Fig. 1*B*, we examine an alternative case, that of cultural evolutionary mismatch in which an authority seeks to shift a population that remains at a high frequency of the zero-sum worldview (one of the solid curves in Fig. 1*B*) toward a positive-sum worldview (i.e., the state with $p^{*}=0$). For example, in multicultural settings, promoting narratives about the economic and social benefits of immigration (e.g., filling labor shortages, cultural enrichment) may aim to counteract zero-sum thinking, where locals perceive immigrants as threats to jobs. Fig. 1*B* shows that the greater the effective conformity $\alpha_{e}$, the more effective the authority’s effort β/|s| must be to push the population to the tipping point where the heterogeneous state ceases to exist.

Overall, if there are cultural authorities advancing a particular worldview for some reasons (personal, material, ideological, religious, etc.), they can push the whole population to the state they prefer if their propaganda is strong enough. Otherwise the population will reach a heterogeneous state with both world views coexisting.

Increasing the strength of conformity with peers, *α*, makes it more difficult to shift the population to a new state. Tight societies are characterized by higher conformity with peers (larger *α*) compared to loose societies. In tight societies, *β* is expected to be higher if the authority is viewed as legitimate and/or traditional. However, if the authority promotes new perspectives and lacks legitimacy, *β* is likely to be smaller. Loose societies have lower levels of conformity with peers (*α*) and less strength of authorities’ persuasion (*β*). The model then predicts poor responsiveness to cultural authorities in loose societies, strong responsiveness in tight societies with legitimate authorities, and intermediate responsiveness in tight societies where cultural authorities promote new perspectives or otherwise lack legitimacy.

In *SI Appendix*, we generalize this model for the case when individuals with different worldviews differ in their assortment coefficients, $\rho_{0}$ and $\rho_{1}$.

As an example, consider a population of initially unbiased individuals who are subject to influence by a new cultural authority promoting a zero-sum world view and providing an environment for assortative interactions among such individuals. The launch of Fox News in the United States in 1996 (36) could be such a case, in that the channel both promoted views about the negative impact of immigration (among other conservative talking-points) and created a sense of community among those who watched it regularly. Particularly since the internet lowered dramatically the costs of information transmission, there have been many initiatives in radio, podcasts, and social media channels that have sought to combine direct cultural persuasion with the creation of audience enclaves of like-minded individuals.

**Fig. 2.**
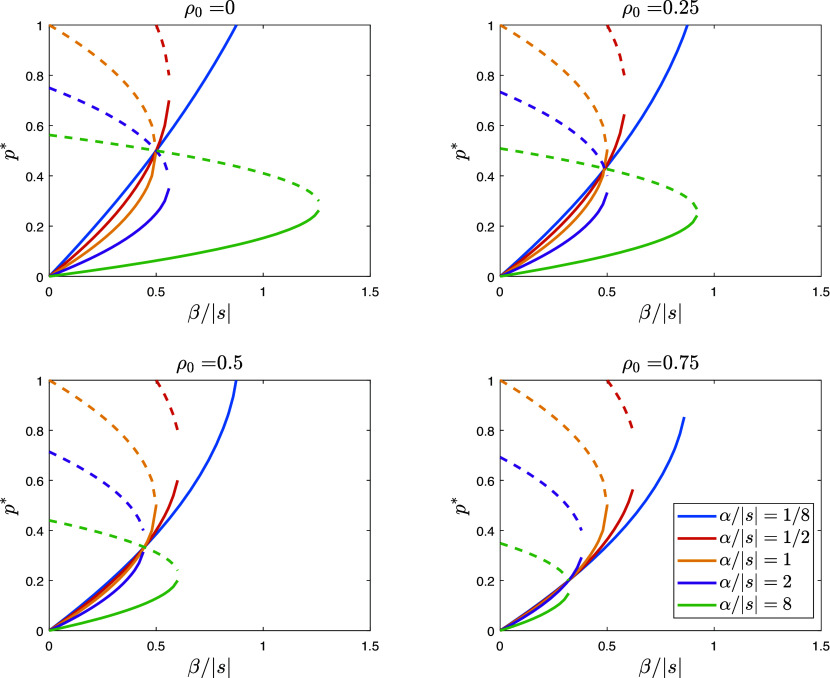
Equilibria of the dynamic Eq. **7** for different values of the scaled parameters β/|s| and α/|s| when the authority promotes a biased zero-sum view and assortative matching at rate $\rho_{0}$ in a population of initially unbiased and randomly matching individuals. Stable equilibria are represented by solid lines, while unstable equilibria are shown by dashed lines. Additionally, there is an equilibrium at p=1, which is stable for β/|s| values to the right of the intersection point of the corresponding solution branch with the horizontal line at p=1.

We can treat those who adopt these worldviews as type 0, with bias $\delta_{0}=\delta$ and assortment coefficient $\rho_{0}=\rho$, while the rest of the population is type 1, with zero bias ($\delta_{1}=0$) and no assortment ($\rho_{1}=0$). Note that if bias were zero for both, type 0 and type 1 would have the same unbiased worldviews. Both types are also influenced by conformity, characterized by the coefficient *α*. Fig. 2 illustrates the effects of parameters on the equilibrium frequency of individuals adopting the zero-sum world view. This figure illustrates that as the assortativeness $\rho_{0}$ of individuals subscribing to the biased worldview increases, it becomes easier for the cultural authority to shift the population toward this worldview. The effect is particularly pronounced in populations with high relative conformity α/|s|, which are more characteristic of tight cultures than loose cultures.

### 1.7. The Incentives for Cultural Authorities.

We have said nothing so far about the incentives for cultural authorities; we have focused on the question whether they can influence equilibrium worldviews, not on why they might want to do so. We now consider whether a new cultural authority might be able profitably to enter a population of individuals who initially are unbiased.

To model this, we make the following assumptions. First, to persuade members of the population to adopt the zero-sum worldview, the new cultural authority sets up a persuasive technology which is financed by the users. An example, already mentioned above, would be the launch of Fox News by Rupert Murdoch’s News Corporation in 1996 as a service provided to an estimated 17 million subscribers to cable TV, rising to 87 million by 2018 (66).

Second, the cost of setting up such a media service increases with the strength of its persuasive technology (*β* in our model), either because more persuasive news and information gathering is expensive, or because advertising to reach viewers is expensive, or both.

Third, the revenue to the cultural authority is proportional to the number of users of the media service. This may be because the media service is able to sell advertising slots at a price proportional to the number of “eyeballs,” or because of carriage fees charged to cable or satellite broadcasting channels, or both.

**Fig. 3.**
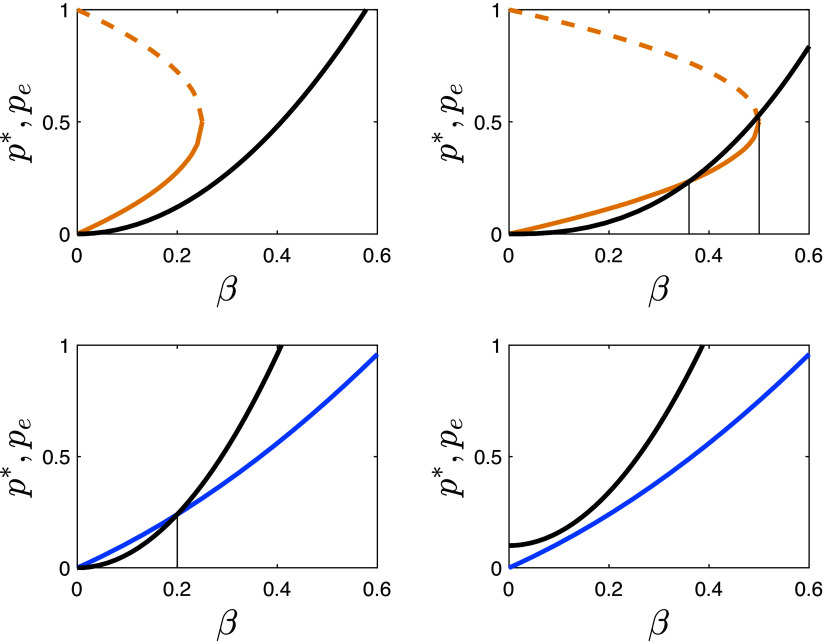
Qualitative depiction of the required frequency of persuaded users, $p_{e}$, needed to finance a persuasive technology with strength *β* (black curves) superimposed on the graph depicting the equilibrium frequency, $p^{*}$, of individuals adopting the worldview promoted by the authority with strength *β* (colored curves). The vertical lines mark the position of the intersection of the two curves.

Together, these assumptions enable us to represent the financial incentives of the cultural authority as an increasing function $m=m_{0}+k\beta^{\eta }$ that, for any given strength of persuasion *β* and assuming η>0, indicates the minimum number of users *m* of the media service that will cover its costs. The proportion of the population that is persuaded is given by βm. Therefore, the proportion $p_{e}$ of the population that must be persuaded to adopt the worldview in order to cover the costs of the media service is given by $p_{e}=\beta m_{0}+k\beta^{1+\eta }$. This persuasion technology function is convex, meaning that each additional increase in *β* requires a progressively larger increase in $p_{e}$. If there are no fixed costs of adopting the technology ($m_{0}=0$) the curve passes through the origin (as in the three graphs in the *Top Left* corner of Fig. 3), while if there are fixed costs ($m_{0}>0$) it has an intercept above the origin (as in the *Bottom Right* graph in that Figure).

In Fig. 3, we superimpose the function $p_{e}\left(\beta \right)$ on the graphs showing the equilibrium frequency $p^{*}$ of users adapting the zero-sum world view promoted by the authority with persuasive strength *β*. In the top two graphs, α/|s|=1 while in the two bottom graphs α/s=1/8.

The technology *β* is “viable,” i.e. it can cover its own expenses, if $p^{*}>p_{e}$. This is the case for all values of *β* in the *Top Left* graph of Fig. 3 but is never the case in the *Bottom Right* graph. In the *Top Right* graph, viable technologies have *β* either smaller than ≈0.36 or above 0.5. In the *Bottom Left* graph, any technology with β≤0.2 is viable.

What these comparisons show is that the likelihood of a cultural authority being able to convince a large part of the population to adopt the zero-sum view increases with factors that shift to the left the curve of equilibrium values of $p^{*}\left(\beta \right)$, or shift to the right the viability curve $p_{e}\left(\beta \right)$ of the authority’s persuasion technology. Fig. 3 shows that factors that shift the equilibrium curve to the left are higher assortment and higher conformity payoffs (*ρ* and *α*). Factors that shift the technology curve to the right include absence of fixed costs of adopting the technology, and low marginal costs of making the technology more persuasive.

### 1.8. Multiple Authorities with Competing Agendas.

The case of a single cultural authority can be generalized to the case of multiple authorities with competing agendas or varying levels of influence. Then the model dynamics can be described by equation

[8] $$\begin{array}{c} \frac{\mathrm{dp}}{\mathrm{dt}}=p\left(1-p\right)\;\left[\underset{\text{natural selection}}{\underbrace{s}}+\underset{\text{conformity}}{\underbrace{\alpha (2p-1)}}\right]+\underset{\text{authority's influence}}{\underbrace{\beta_{0}\left(1-p\right)-\beta_{1}p,}} \end{array}$$

where $\beta_{0}$ and $\beta_{1}$ are the net strengths of the authorities promoting worldviews θ=0 and θ=1, respectively.

If the authorities’ strengths are the same, $\beta_{0}=\beta_{1}=\beta$, then the authorities’ influence term becomes β(1−2p), which is similar to the conformity term but with an opposite sign. This means that the presence of equally strong persuasive technologies effectively decreases the conformity from *α* to α−β. In this sense, authorities act as anticonformity promoters. Thus, if the net effect of authorities is less than that of conformity, i.e., if β<α, the conclusions from the section on conformity apply directly. However, if β>α, then evolutionary outcomes depend on the relative values of β−α and the strength of selection |s|. If β−α<|s|, the system evolves to a homogeneous state with the higher payoff: p=1 if s>0 or p=0 if s<0. However, if the effect of authorities is very strong, so that β−α>|s|, the system evolves to a heterogeneous state where both worldviews coexist and $p^{*}=\frac{1}{2}\left(1+\frac{s}{\beta -\alpha }\right)$.

**Fig. 4.**
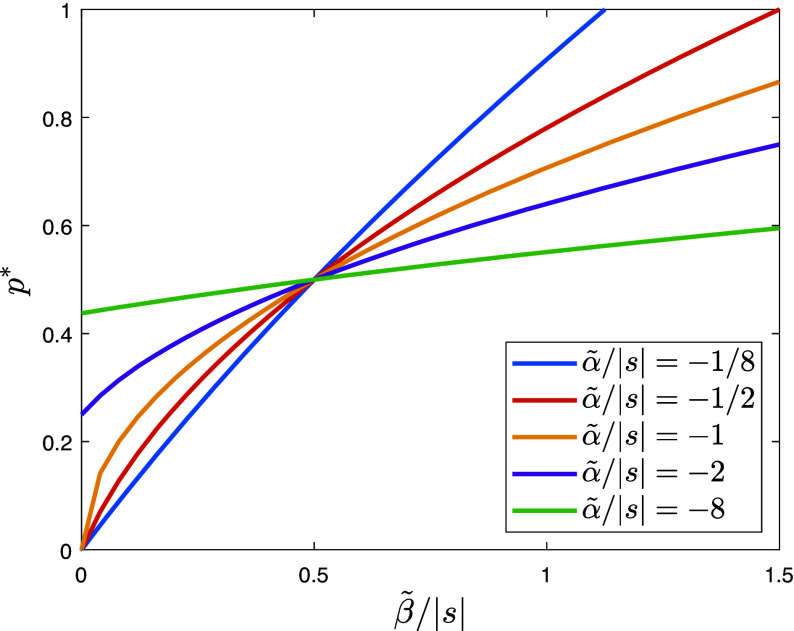
Stable equilibria of the dynamic Eq. **8** for different values of the scaled parameters $\tilde{\beta }/\left|s\right|$ and $\tilde{\alpha }/\left|s\right|$ in the model with $\beta_{0}>\alpha$. Additionally, there is an equilibrium at p=1, which is stable for $\tilde{\beta }/\left|s\right|$ values to the right of the intersection point of the corresponding solution branch with the horizontal line at p=1.

If the authorities’ strengths differ, say $\beta_{0}>\beta_{1}$, we can rewrite the last term in Eq. **10** as $\beta_{1}\left(1-2p\right)+\left(\beta_{0}-\beta_{1}\right)\left(1-p\right)$. This implies that Eq. **10** reduces to Eq. **9** if we redefine *α* as $\tilde{\alpha }=\alpha -\beta_{1}$ and *β* as $\tilde{\beta }=\beta_{0}-\beta_{1}$. Essentially, if $\alpha >\beta_{1}$, the conformity parameter *α* is reduced by the strength of the less strong technology, while the persuasion strength term is replaced by the difference in strengths. In Fig. 4, this results in a shift to a higher curve of equilibria and a shift to the left along the horizontal axis. These two shifts have opposite effects on the equilibrium frequency $p^{*}$ of individuals with worldview γ=0, so the net effect will depend on the balance of parameters.

However, if the strength of the weaker authority exceeds the effect of conformity, i.e., if $\alpha <\beta_{1}$, there is always only one stable equilibrium: at an intermediate *p*, describing a heterogeneous population for relatively small values of $\tilde{\beta }$, or at p=1, describing a homogeneous population for relatively large values of $\tilde{\beta }$.

To understand the relation between Figs. 1 and 4, it should be noted that Fig. 1 describes the possible equilibrium proportions of the two types as a function of different levels of population conformity and the persuasiveness of the authority, where there is only one authority. Fig. 4 adapts this to a setting of more than one authority and shows the possible equilibrium proportions of types as a function of the net conformity (gross conformity of the population net of the offsetting effect of the weaker authority) and the net persuasiveness (the persuasiveness of the stronger authority net of the offsetting effect of the weaker authority).

## 2. Discussion

Individuals’ predispositions to hold beliefs about the nature of the world—especially whether they perceive it as a zero-sum or a positive-sum environment—can profoundly influence a range of personal and societal outcomes, including trust, cooperation, policy preferences, and overall well-being. Over the past several decades, these influences have attracted widespread attention from researchers in economics, psychology, and other social sciences, significantly shaping discussions around human behavior and decision-making (1, 10). However, the underlying factors that shape these differing worldviews have received far less attention—especially from researchers using mathematical modeling, a powerful approach given the complexity of the factors involved (with the exception of ref. 8).

In this study, we employ mathematical modeling to investigate the evolution of worldviews within a population, focusing on how natural and cultural selection shape perceptions of the payoff environment as either zero-sum or positive-sum. Our model integrates the influence of material payoffs with the effects of assortative matching and social influence exerted by peers and by cultural authorities. Specifically, we analyze pairwise interactions within a heterogeneous population composed of individuals biased toward believing in a zero-sum world, who tend to invest more in competition, and those biased toward believing in a positive-sum world, who are more inclined to invest in cooperation.

With random matching and in the absence of social influence, if the bias of the two types of individuals is of the same magnitude, and if worldviews spread in proportion to the relative payoffs of those holding them, there is a unique equilibrium in which selection favors the bias toward the more probable environment. With unequal bias, it favors the bias whose overall probability of error is lower. This is the precise sense in which we can say that the objective state of the environment favors more accurate worldviews.

With assortative matching based on bias type, we find that the positive-sum worldview can persist even when the positive-sum environment is objectively less common (below 50%) than the competitive environment. This highlights a counterintuitive dynamic: Social interaction structures alone can sustain beneficial yet objectively inaccurate worldviews. This finding is conceptually reminiscent—though driven by a very different mechanism—of results from evolutionary biology, where genetic relatedness can favor cooperative strategies over defection (56), but see ref. 67).

In the presence of conformity with peers, the population may no longer evolve toward a unique equilibrium; instead, the outcome can depend on the initial frequencies of the two worldviews. Consequently, conformity can promote the persistence of inaccurate beliefs following environmental changes, resulting in a cultural evolutionary mismatch.

Finally, we examine the influence of cultural authorities capable of shaping worldviews, with a particular focus on authorities who promote a zero-sum worldview in a population of individuals who are initially unbiased. Our analysis reveals that the presence of such authorities can lead to multiple equilibria, characterized by either homogeneous or heterogeneous worldview distributions, depending on the parameter values. Notably, our model shows that cultural authorities can trigger tipping points. Small incremental increases in the strength of persuasive messaging can lead to sudden and complete adoption of a suboptimal worldview, highlighting the significant potential influence of even modest shifts in media or authoritative messaging.

When both types of worldviews exhibit similar levels of assortative matching, stronger assortativity hinders the authority’s efforts to spread the zero-sum worldview. However, if authorities actively create conditions that enhance asymmetric assortativity—such as through specialized social media platforms tailored to individuals already inclined toward the worldview they promote—stronger assortativity has the opposite effect. Moreover, our analysis explicitly demonstrates how cultural authorities can find economic incentives to amplify biased worldviews by intentionally cultivating assortative enclaves. This economically driven reinforcement of biases provides an explanation for the persistent and profitable propagation of misinformation and polarized narratives in contemporary societies. Additionally, the competition between multiple cultural authorities can significantly diminish conformity pressures, potentially reducing cultural rigidity and mitigating evolutionary mismatches. This insight suggests practical policy implications for media regulation or educational reforms aiming to counteract polarization.

Our results predict greater responsiveness to both entrepreneurs and authorities, and consequently a higher likelihood of cultural evolutionary mismatch, in tight societies, where strong social norms amplify conformity and susceptibility to external influence, compared to loose societies, which buffer such effects through norm flexibility. Loose cultures encourage more experimentation, yet the same tolerance that supports diversity also weakens the peer and authority pressures necessary to turn innovations into cascades. Consequently, entrepreneurs frequently launch innovations, but these tend to remain niche; loose societies are, in a sense, “nimble at the edges, sticky at the center.” Tight cultures exhibit the opposite pattern: Deviation is rare, but once a legitimate authority promotes a new norm, strong conformity rapidly spreads it across society as seen in East Asia’s quick adoption of COVID-19 mask mandates or China’s Cultural Revolution.

Our findings also speak directly to current debates about political polarization and echo chambers in media environments. Specifically, the model elucidates how modern media platforms, by combining persuasive messaging with assortative audience targeting, can significantly enhance polarization and cultural evolutionary mismatch.

There are many historical and contemporary examples where political, cultural, and religious leaders have successfully framed social, economic, or political issues in zero-sum terms, often convincing a large proportion of the population that gains for one group necessarily mean losses for another. This strategy is particularly effective in times of economic uncertainty, cultural change, or geopolitical tension. For example, Donald Trump’s rhetoric has frequently framed economic and immigration policies in zero-sum terms, such as arguing that jobs taken by immigrants come at the direct expense of native-born citizens or that international trade agreements unfairly benefit other countries at America’s expense (33). During the 2016 Brexit Campaign in the United Kingdom, the “Take Back Control” slogan, subsequently taken up by the UK Government, implied that resources sent to the European Union were a loss for Britain, rather than part of a mutually beneficial system (68).

Zero-sum rhetoric can be found at all points of the political spectrum. In early 2025 Senator Bernie Sanders responded to Elon Musk’s call for more H-1B visas for tech workers as follows: “The main function of the H-1B visa program and other guest worker initiatives is not to hire “the best and the brightest,” but rather to replace good-paying American jobs with low-wage indentured servants from abroad...The cheaper the labor they hire, the more money the billionaires make.” (69). In a different dimension of policy, the charity Oxfam released a report in 2023 claiming that “Oxfam believes that, as a starting point, the world should aim to halve the wealth and number of billionaires between now and 2030, both by increasing taxes on the top 1% and adopting other billionaire-busting policies. This would bring billionaire wealth and numbers back to where they were just a decade ago in 2012. The eventual aim should be to go further, and to abolish billionaires altogether, as part of a fairer, more rational distribution of the world’s wealth” (70).

Arguments stressing the positive-sum nature of society have also been deployed, apparently to good effect, at certain crisis moments in society, such as the start of the COVID pandemic (31), the Russian invasion of Ukraine (71) and outbreaks of social unrest (72). However, the long-run impact of such interventions remains to be demonstrated. More generally, research on interventions specifically designed to shift individuals’ perspectives from a zero-sum to a positive-sum worldview remains scarce.

The zero-sum perspective is intuitive and exploits loss aversion, making it a powerful political tool. It is easier to communicate and mobilize support around than are nuanced, long-term positive-sum arguments. Moreover, individuals experiencing economic hardship or social change are more likely to adopt zero-sum thinking, making them particularly receptive to such messages. In terms of our models, this suggests that messaging promoting the zero-sum worldview may be characterized by a larger *β* compared to messaging that advances a positive-sum worldview.

A natural question to ask is whether, if societies can become stuck in a cultural evolutionary mismatch, there is anything that political leadership can do to improve outcomes. Despite the growing literature on the determinants and effects of zero-sum mindsets, this is a question that has not so far convincingly been addressed. As Andrews Fearon and Götz (1) put it, “In line with our understanding of the zero-sum mindset as a relatively stable individual difference, it may be unreasonable to assume that such a belief can be meaningfully altered...using many of the light-touch methods commonly employed in experimental psychology.” This does not of course mean that it cannot be altered at all, rather that efforts to counter zero-sum thinking need to be ambitious in scope. In their review of ten years of empirical research, Matsunaga et al. (10) assert that because zero-sum thinking is “not just a general worldview, but also a situational bias triggered by perceived threats, competition, and group status,” it is “a bias with potential solutions from behavioral science.”

Most of the studies that aim to promote cooperation via behavioral interventions and have been subject to randomized controlled trials are not like this, however (73). They rely typically on reminders to subjects of the presence of norms, which fall far short of what we have described as changes in an entire world view. Although a number of social and political initiatives, such as the Truth and Reconciliation Commission established after the end of apartheid in South Africa, have sometimes been considered as ambitious attempts to change the worldview of whole societies, we are not aware of any rigorous evaluations of such attempts.

Finally, we should note that to achieve tractability and communicate intuition, our model makes some important but reasonable simplifications. We do not propose our model as a detailed description of what occurs in the cases just described, but rather as a clarification of the mechanisms that may be at work. In the case of the abolition of apartheid, for example, cultural authorities were determined to persuade the population that the emancipation of the black population did not have to mean equivalent losses for the white population, although many of the white population were initially convinced otherwise. Understanding these mechanisms will be easier if we are explicit about the simplifications we impose.

First, we assume a well-mixed population. However, in reality, individuals are embedded in social networks, whose structure can influence the dynamics of worldviews.

Second, we assume only two worldviews to be competing with each other. The presence of three or more simultaneously competing worldviews would be a more realistic assumption but would be harder to model in an intuitively comprehensible way.

Third, we assume worldviews spread if they lead individuals to have higher utilities, regardless of whether these are material payoffs or psychological benefits. This is reasonable despite well-known exceptions, like the famously depressive movies made by the famously depressive director Ingmar Bergman which have helped to spread his famously depressive worldview (74).

Fourth, the interactions in our model have the property that optimal strategies for individuals are independent of what other players do (though their payoffs are of course dependent on other players’ actions). This means we can avoid having to consider higher-order beliefs, which would greatly complicate the model for little additional insight.

We have assumed that the environment is uncorrelated over time. Alternatively, one could posit periods (or ‘epochs’) of environmental stability by modeling the environment as a two-state Markov chain with constant transition probabilities. Longer spells of stable conditions would allow selection to operate over extended periods before the environment shifts, thereby influencing the duration and extent of cultural mismatch. We leave a detailed exploration of this important question for future work.

Finally, if cultural authorities are driven by economic profits, we must first specify their revenue source. In typical economic models (e.g., those of media firms), this revenue comes from the audience, thereby reducing each audience member’s material payoff. Here, however, we assume that zero-sum converts do not bear any extra payoff loss. Although not fully general, this assumption is often empirically plausible. For example, nearly all cable subscribers in the United States pay for Fox News-through both subscriptions and ad views-whether or not they adopt its zero-sum worldview (66).

Our findings suggest that while the evolution of worldviews under natural selection favors some alignment with reality, less accurate worldviews can still prevail over more accurate ones when reinforced by assortativity, social pressure to conform, and the influence of cultural authorities. The findings remain of relevance in a wider range of contexts than the admittedly simple setting of our mathematical model.

## Supplementary Material

Appendix 01 (PDF)

## Data Availability

There are no data underlying this work.

## References

[1] P. Andrews Fearon, F. M. Götz, The zero-sum mindset. J. Pers. Soc. Psychol. **127**, 758–795 (2024). 39570690 10.1037/pspa0000404

[2] J. K. Rilling , Neural correlates of social cooperation and non-cooperation as a function of psychopathy. Biol. Psychiatry **61**, 1260–1271 (2007). 17046722 10.1016/j.biopsych.2006.07.021

[3] X. Zhu, Z. Li, Seeking or giving help? Linkages between the dark triad traits and adolescents’ help seeking and giving orientations: The role of zero-sum mindset Pers. Individ. Differ. **236**, 113031 (2025).

[4] J. Berg, J. Dickhaut, K. McCabe, Trust, reciprocity, and social history. Games Econ. Behav. **10**, 122–142 (1995).

[5] P. Seabright, “Is cooperation habit-forming?” in *The Environment and Emerging Development Issues*, P. Dasgupta, K. G. Mäler, Eds. (Clarendon Press, 1997).

[6] E. Fehr, S. Gächter, Cooperation and punishment in public goods experiments. Am. Econ. Rev. **90**, 980–994 (2000).

[7] N. Ashraf, I. Bohnet, N. Piankov, Decomposing trust and trustworthiness. Exp. Econ. **9**, 193–208 (2006).

[8] A. Bergeron, J. P. Carvalho, J. Henrich, N. Nunn, J. L. Weigel, *Zero-Sum Thinking, The Evolution of Effort-Suppressing Beliefs, and Economic Development* (National Bureau of Economic Research, Working Paper 31663, 2023).

[9] J. Różycka-Tran, P. Boski, B. Wojciszke, Belief in a zero-sum game as a social axiom: A 37-nation study. J. Cross-Cult. Psychol. **46**, 525–548 (2015).

[10] L. H. Matsunaga, J. Petersen, T. Aoki, C. Faiad, The science of zero-sum thinking: A scoping review of 10 years of empirical research. Dyn. Asymmetric Confl. **2**, 1–21 (2024).

[11] S. Chinoy, N. Nunn, S. Sequeira, S. Stantcheva, *Zero-Sum Thinking and the Roots of U.S. Political Divides* (National Bureau of Economic Research, Working Paper 31688, 2023).

[12] M. I. Norton, S. R. Sommers, Whites see racism as a zero-sum game that they are now losing. Perspect. Psychol. Sci. **6**, 215–218 (2011). 26168512 10.1177/1745691611406922

[13] C. S. Dweck, Mindset: The New Psychology of Success (Random House, New York, 2006).

[14] S. Chinoy, N. Nunn, S. Sequeira, S. Stantcheva, Zero-sum thinking and the roots of U.S. political differences. arXiv [Preprint] (2024). https://www.aeaweb.org/conference/2025/program/paper/7ffAAan4 (Accessed 19th July 2025).

[15] M. E. Koltko-Rivera, The psychology of worldviews. Rev. Gen. Psychol. **8**, 3–58 (2004).

[16] R. Mifsud, G. Sammut, Worldviews and the role of social values that underlie them. PLoS One **18**, e0288451 (2023). 37494357 10.1371/journal.pone.0288451 PMC10370735

[17] M. Gelfand, S. Gavrilets, N. Nunn, Norm dynamics: Interdisciplinary perspectives on social norm emergence, persistence, and change. Annu. Rev. Psychol. **75**, 341–378 (2024). 37906949 10.1146/annurev-psych-033020-013319

[18] A. Nilsson, “The psychology of worldviews: Toward a non-reductive science of personality,” PhD thesis, Lund University, Lund, Sweden (2013).

[19] A. Nilsson, A non-reductive science of personality, character, and well-being must take the person’s worldview into account. Front. Psychol. **5**, 961 (2014). 25221538 10.3389/fpsyg.2014.00961 PMC4147830

[20] V. Calabuig, G. Olcina, F. Panebianco, Culture and team production. J. Econ. Behav. Organ. **149**, 32–45 (2018).

[21] S. Gavrilets, Coevolution of actions, personal norms, and beliefs about others in social dilemmas. Evol. Hum. Sci. **3**, e44 (2021). 37588544 10.1017/ehs.2021.40 PMC10427329

[22] N. V. Peale, The Power of Positive Thinking (Prentice Hall, 1952).

[23] J. A. Fletcher, M. Doebeli, A simple and general explanation for the evolution of altruism. Proc. R. Soc. Lond. B **276**, 13–19 (2009). 10.1098/rspb.2008.0829 PMC2614248 18765343

[24] I. Alger, J. W. Weibull, Evolution and Kantian morality. Games Econ. Behav. **98**, 56–67 (2016).

[25] H. Tajfel, “Social categorization, social identity, and social comparisons” in *Differentiation Between Social Groups: Studies in the Social Psychology of Intergroup Relations*, H. Tajfel, Ed. (Academic Press, London, 1978), pp. 27–60.

[26] R. Axelrod, The dissemination of culture: A model with local convergence and global polarization. J. Confl. Res. **41**, 203–226 (1997).

[27] N. Nunn, On the dynamics of human behavior: The past, present, and future of culture, conflict, and cooperation. AEA Pap. Proc. **112**, 15–37 (2014).

[28] N. Nunn, “On the causes and consequences of cross-cultural differences: An economic perspective” in *Handbook of Advances in Culture and Psychology*, M. J. Gelfand, C. Y. Chiu, Y. Y. Hong, Eds. (Oxford University Press, Oxford, 2022), pp. 125–188.

[29] M. J. Gelfand, Cultural evolutionary mismatches in response to collective threat. Curr. Dir. Psychol. Sci. **30**, 5401–5409 (2021).

[30] B. Flynn, Bossing It: For Bridget Flynn We’re All in This Together (Little Black Book, 2024).

[31] A. Guterres, We Are All in This Together: Human Rights and COVID-19 Response and Recovery (United Nations, 2020).

[32] E. Okoro, We Are All in This Together (Financial Times, 2024).

[33] S. Kohn, Nothing Donald Trump Says on Immigration Holds Up (Time Magagement, 2016).

[34] M. Gentzkow, J. M. Shapiro, What drives media slant? Evidence from U.S. daily newspapers Econometrica **78**, 35–71 (2010).

[35] R. Durante, B. Knight, Partisan control, media bias, and viewer responses: Evidence from Berlusconi’s Italy. J. Eur. Econ. Assoc. **10**, 451–481 (2012).

[36] G. J. Martin, A. Yurukoglu, Bias in cable news: Persuasion and polarization. Am. Econ. Rev. **107**, 2565–2599 (2017).

[37] M. J. Gelfand , Differences between tight and loose cultures: A 33-nation study. Science **332**, 1100–1104 (2011). 21617077 10.1126/science.1197754

[38] R. L. Trivers, The elements of a scientific theory of self-deception. Ann. N. Y. Acad. Sci. **907**, 114–131 (2000). 10818624 10.1111/j.1749-6632.2000.tb06619.x

[39] P. Schwardmann, J. van der Weele, Deception and self-deception. Nat. Hum. Behav. **3**, 1055–1061 (2019). 31358973 10.1038/s41562-019-0666-7

[40] I. Esponda, D. Pouzo, Berk-Nash equilibrium: A framework for modeling agents with misspecified models. Econometrica **84**, 1093–1130 (2016).

[41] F. Massari, J. Newton, Learning and equilibrium in misspecified models. SSRN [Preprint] (2020). https://ssrn.com/abstract=3473630 (Accessed 20 July 2025).

[42] G. M. Foster, Peasant society and the image of limited good. Am. Anthropol. **67**, 293–315 (1965).

[43] B. Gershman, The economic origins of the evil eye belief. J. Econ. Behav. Organ. **110**, 119–144 (2015).

[44] D. Hume, *A Treatise of Human Nature* L. A. Selby-Bigge, Ed. (Oxford: Clarendon Press, Oxford, ed. 1888 1739), pp. Book III, Part II, Section V, p. 520.

[45] A. Smith, *An Inquiry into the Nature and Causes of the Wealth of Nations* (W. Strahan and T. Cadell, London) (1776).

[46] T. Kuran, W. H. Sandholm, Cultural integration and its discontents. Rev. Econ. Stud. **75**, 201–228 (2008).

[47] V. Calabuig, G. Olcina, F. Panebianco, The erosion of personal norms and cognitive dissonance. Appl. Econ. Lett. **23**, 1265–1268 (2016).

[48] V. Calabuig, G. Olcina, F. Panebianco, The dynamics of personal norms and the determinants of cultural homogeneity. Ration. Soc. **29**, 322–354 (2017).

[49] G. Olcina, F. Panebianco, Y. Zenou, Conformism, social pressure, and the dynamics of integration. J. Econ. Behav. Organ. **220**, 279–304 (2024).

[50] S. Gavrilets, D. Tverskoi, A. Sanchez, Modeling social norms: An integration of the norm-utility approach with beliefs dynamics. Philos. Trans. R. Soc. Lond. B **379**, 20230027 (2024). 38244599 10.1098/rstb.2023.0027 PMC10799741

[51] P. D. Taylor, L. B. Jonker, Evolutionary stable strategies and game dynamics. Math. Biosci. **40**, 145–156 (1978).

[52] P. Schuster, K. Sigmund, Replicator dynamics. J. Theor. Biol. **100**, 533–538 (1983).

[53] J. Hofbauer, K. Sigmund, The Theory of Evolution and Dynamical Systems (Cambridge University Press, Cambridge, 1988).

[54] W. H. Sandholm, Population Games and Evolutionary Dynamics (MIT Press, Cambridge, Massachusetts, 2010).

[55] R. McElreath, R. Boyd, Mathematical Models of Social Evolution. A Guide for the Perplexed (Chicago University Press, Chicago, 2007).

[56] W. Hamilton, The genetical evolution of social behaviour I and II. J. Theor. Biol. **7**, 1–52 (1964). 5875341 10.1016/0022-5193(64)90038-4

[57] R. Bond, P. B. Smith, Culture and conformity: A meta-analysis of studies using Asch’s (1952b, 1956) line judgment task. Psychol. Bull. **119**, 111–137 (1996).

[58] J. Henrich, The Secret of Our Success (Princeton University Press, Princeton, NJ, 2015).

[59] P. J. Richerson, S. Gavrilets, F. B. M. de Waal, Modern theories of human evolution foreshadowed by Darwin’s Descent of Man. Science **372**, eaba3776 (2021). 34016754 10.1126/science.aba3776

[60] S. Gavrilets, Social Influence and the Logic of Collective Action (Princeton University Press, Princeton, NJ, 2025).

[61] W. Güth, M. Yaari, An evolutionary approach to explain reciprocal behavior in a simple strategic game. Explain. Process. Chang. Approach. Evol. Econ. **24**, 23–34 (1992).

[62] E. A. Ok, F. Vega-Redondo, On the evolution of individualistic preferences: An incomplete information scenario. J. Econ. Theory **97**, 231–254 (2001).

[63] R. Boyd, P. J. Richerson, Culture and the Evolutionary Process (University of Chicago Press, Chicago, 1985).

[64] M. G. Bulmer, Theoretical Evolutionary Ecology (Sinauer Associates, Sunderland, MA, 1994).

[65] F. M. Bass, A new product growth model for consumer durables. Manag. Sci. **15**, 215–227 (1969).

[66] Wikipedia contributors, Fox News — Wikipedia. https://en.wikipedia.org/w/index.php?title=Fox_News&oldid=1272631500. Accessed 1 February 2025.

[67] Y. Dong, S. Gavrilets, C. Z. Qin, B. Zhang, Kinship can hinder cooperation in heterogeneous populations. J. Econ. Behav. Organ. **219**, 231–243 (2024).

[68] H Government, Taking Back Control of Our Borders, Money and Laws While Protecting Our Economy, Security and Union (UK Government, Policy paper, 2018).

[69] A. Stanton, Bernie Sanders Sides with MAGA on H-1B Debate (Newsweek, 2025).

[70] M.-B. Christensen, C. Hallum, A. Maitland, Q. Parrinello, C. Putaturo, Survival of the richest: How we must tax the super-rich now to fight inequality (2023). https://policy-practice.oxfam.org/resources/survival-of-the-richest-how-we-must-tax-the-super-rich-now-to-fight-inequality-621477/. Accessed 20 July 2025.

[71] G. Leitlande, Societal resolve amid war: Lessons from Ukraine’s response to the Russian invasion. Obrana Strateg. **24**, 99–111 (2024).

[72] D. Cameron, We Are All in This Together (Ipswich Conservation, 2011).

[73] E. L. Paluck, H. Shepherd, P. M. Aronow, Changing climates of conflict: A social network experiment in 56 schools. Proc. Natl. Acad. Sci. U.S.A. **113**, 566–571 (2016). 26729884 10.1073/pnas.1514483113 PMC4725542

[74] T. Branigan, Even I Think My Films Are Depressing, Admits Ingmar Bergman (The Guard, 2004).

