# Supplementary material for: The evolution of zero-sum and positive-sum worldviews
Source: Proc Natl Acad Sci U S A. 2025 Aug 5;122(32):e2504339122. doi: 10.1073/pnas.2504339122 (PMC12358829; doi:10.1073/pnas.2504339122)
Supplement: Supplementary file 1 — Appendix 01 (PDF) [file pnas.2504339122.sapp.pdf]

The evolution of zero-sum and positive-sum world-views

Sergey Gavrilets and Paul Seabright

Supplementary information

Best response actions and payoffs given beliefs. The payoff in state e of an individual i interacting with an individual j can be written as

$$\pi_i(e) = (1-e)\underbrace{b_0(x_i - x_j)}_{\text{benefit if e=0}} - \underbrace{\frac{1}{2}c_0 x_i^2}_{\text{cost of investing in x}} + e\,\underbrace{b_1(y_i + y_j)}_{\text{benefit if e=1}} - \underbrace{\frac{1}{2}c_1 y_i^2}_{\text{cost of investing in y}}, \qquad \text{[S1]}$$

To facilitate analysis, we define the composite parameters

$$r_0 = \frac{b_0^2}{2c_0}, \quad r_1 = \frac{b_1^2}{2c_1},$$

which measure the payoffs of player 0 in environment 0 and player 1 in environment 1, respectively, when they hold the correct belief about the state of the environment, while their opponent holds an incorrect belief (as discussed below).

Given the state of the environment and the agents' beliefs about it, finding the Nash equilibrium efforts is straightforward. Using equation (S1), by equating the derivatives $d\pi_i(e)/dx_i$ and $d\pi_j(e)/dx_j$ to zero and solving the resulting system of linear algebraic equations, one find the following.

- If both players believe that $e = 0$, they both invest in competition. Their equilibrium efforts and payoffs are:

$$x_i = \frac{b_0}{c_0}, \quad y_i = 0, \quad \pi_i = F_{00} \equiv -r_0.$$

- If both players believe that $e = 1$, they both invest in cooperation. Their equilibrium efforts and payoffs are:

$$x_i = 0, \quad y_i = \frac{b_1}{c_1}, \quad \pi_i = F_{11}(e) \equiv r_1(4e-1).$$

- If player i believes that $e = 0$ and invests in competition, while player j believes that $e = 1$ and invests in cooperation, then:

$$x_i = \frac{b_0}{c_0}, \quad y_i = 0, \quad \pi_i = F_{01}(e) \equiv r_0 + 2(r_1 - r_0)e, x_j \quad = 0, \quad y_j = \frac{b_1}{c_1}, \quad \pi_j = F_{10}(e) \equiv -2r_0 - r_1 + 2(r_0 + r_1)e.$$

Note that players' efforts do not depend on the actual state of the environment, whereas their payoffs (except for the first case) do. Note also that we introduced variables F for payoffs which will be used below.

In general, not all Nash equilibria are equilibria of replicator dynamics, and not all equilibria of replicator dynamics are Nash equilibria (54, 75). In our case, however, the best response is independent of other players' actions, and the long-term outcome of myopic optimization does converge to the Nash equilibrium.

Expected payoffs. To find expected payoffs of two types of players we need to account for environmental stochasticity and the possibility of errors in evaluation the environmental state. For mathematical simplicity, we assume that agents of the same type share identical beliefs about the current environment, meaning they are collectively either right or wrong. Agents of different types can have different beliefs about the current environment.

Assuming random matching,

$$\pi_0 = \underbrace{p\left[q \times F_{00} + (1-q) \times \left((1-\delta)^2 F_{11}(1) + (1-\delta)\delta(F_{10}(1) + F_{01}(1)) + \delta^2 F_{00}\right)\right]}_{0\times 0 \text{ matching}} \qquad \text{[S2a]}$$

$$+ \underbrace{(1-p)\ \left[q \times (\delta F_{01} + (1-\delta)F_{00}) + (1-q) \times (\delta F_{01}(1) + (1-\delta)\, F_{11}(1))\right]}_{0\times 1 \text{ matching}},$$

$$\pi_1 = \underbrace{p\left(q \times ((1-\delta)\, F_{00} + \delta F_{10}(0)) + (1-q) \times (\delta F_{10}(1) + (1-\delta)\, F_{11}(1))\right)}_{1\times 0 \text{ matching}} \qquad \text{[S2b]}$$

$$+ \underbrace{(1-p)\left(q \times ((1-\delta)^2 F_{00} + (1-\delta)\delta(F_{10}(0) + F_{01}(0)) + \delta^2 F_{11}(0)) + (1-q) \times F_{11}(1)\right)}_{1\times 1 \text{ matching}}$$

Simplifying the difference of the payoffs we find that

$$s \equiv \pi_0 - \pi_1 = \delta(2q-1)(r_0 + r_1). \qquad \text{[S3]}$$

Similar calculations can be done in the case that the two types differ in the probability δ_i of making an error. In this case,

$$\pi_0 = \underbrace{p\left[q \times F_{00} + (1-q) \times \left((1-\delta_0)^2 F_{11}(1) + (1-\delta_0)\delta_0(F_{10}(1) + F_{01}(1)) + \delta_0^2 F_{00}\right)\right]}_{0 \times 0 \text{ matching}} \ldots \quad \text{[S4a]}$$

$$+ \underbrace{(1-p)\ \ [q \times (\delta_1 F_{01}(0) + (1-\delta_1) F_{00}) + (1-q) \times (\delta_0 F_{01}(1) + (1-\delta_0) F_{11}(1))]}_{0 \times 1 \text{ matching}},$$

$$\pi_1 = \underbrace{p\,(q \times ((1-\delta_1) F_{00} + \delta_1 F_{10}(0)) + (1-q) \times (\delta_0 F_{10}(1) + (1-\delta_0) F_{11}(1)))}_{1 \times 0 \text{ matching}} \quad \text{[S4b]}$$

$$+ \underbrace{(1-p)\left(q \times ((1-\delta_1)^2 F_{00} + (1-\delta_1)\delta_1(F_{10}(0) + F_{01}(0)) + \delta_1^2 F_{11}(0)) + (1-q) \times F_{11}(1)\right)}_{1 \times 1 \text{ matching}}$$

One finds that

$$s \equiv \pi_0 - \pi_1 = [q\delta_1 - (1-q)\delta_0](r_0 + r_1). \quad \text{[S5]}$$

Note the values $q\delta_1$ and $(1-q)\delta_0$ represent the overall error rates for type 1 and type 0, respectively, accounting for the frequencies of the different environmental states.

If matching is assortative rather than random,

$$\pi_0 = \underbrace{(\rho_0 + p(1-\rho_0))\left[q \times F_0 + (1-q) \times \left((1-\delta_0)^2 F_{11}(1) + (1-\delta_0)\delta_0(F_{10}(1) + F_{01}(1)) + \delta_0^2 F_{00}\right)\right]}_{0 \times 0 \text{ matching}} \quad \text{[S6a]}$$

$$+ \underbrace{(1-\rho_0 - p(1-\rho_0))\,[q \times (\delta_1 F_{01}(0) + (1-\delta_1) F_{00}) + (1-q) \times (\delta_0 F_{01}(1) + (1-\delta_0) F_{11}(1))]}_{0 \times 1 \text{ matching}},$$

$$\pi_1 = \underbrace{(1-\rho_1 - (1-p)(1-\rho_1))\,[q \times ((1-\delta_1) F_{00} + \delta_1 F_{10}(0)) + (1-q) \times (\delta_0 F_{10}(1) + (1-\delta_0) F_{11}(1))]}_{1 \times 0 \text{ matching}} \quad \text{[S6b]}$$

$$+ \underbrace{(\rho_1 + (1-p)(1-\rho_1))\left[q \times ((1-\delta_1)^2 F_{00} + (1-\delta_1)\delta_1(F_{10}(0) + F_{01}(0)) + \delta_1^2 F_{11}(0)) + (1-q) \times F_{11}(1)\right]}_{1 \times 1 \text{ matching}}$$

If $\delta_0 = \delta_1 = \delta$ and $\rho_0 = \rho_1 = \rho$, the difference in payoffs is

$$\pi_0 - \pi_1 = \underbrace{(r_0 + r_1)\delta(2q-1)}_{\textbf{selection}} - \underbrace{2\delta\rho[r_0 q + r_1(1-q)]}_{\text{assortment}}. \quad \text{[S7]}$$

The second term is always negative. This implies that in the symmetric case assortment decreases the range of parameter values leading to positive s and the spread of the zero-sum world view.

In the general case,

$$\pi_0 - \pi_1 = \underbrace{[q\delta_1 - (1-q)\delta_0](r_0 + r_1)}_{\textbf{selection}} - \underbrace{2[r_0 q\delta_1 + r_1(1-q)\delta_0]\ [(1-p)\rho_0 + p\rho_1]}_{\text{assortment}}. \quad \text{[S8a]}$$

The last term is just the average coefficient of assortment in the population $\bar{\rho}(p) = (1-p)\rho_0 + p\rho_1$ which depends on p. Correspondingly, we can rewrite equation (S8a) as

$$\pi_0 - \pi_1 = s - 2\sigma\ \bar{\rho}(p), \quad \text{[S8b]}$$

with the obvious meaning of s and σ.

Utilities due to conformity and assortment. We assume that the two types differ in their conformity and assortment parameters. An individual of type 0 expects a total utility due to conformity

$$u_0^c = \alpha_1 \times \ \left[\text{prob. of meeting another type } 0\right].$$

Under the assortative matching, with probability ρ_0 the individual meets another type 0 for sure, and with probability $(1-\rho_0)$ the partner is chosen at random from the population proportions. Hence,

$$\text{prob. of meeting type } 0 = \rho_0 + (1-\rho_0)p.$$

Therefore,

$$u_0^c = \alpha_0[\rho_0 + (1-\rho_0)p].$$

Similarly, an individual of type 1 earns

$$u_1^c = \alpha_1 \times \left[\text{prob. of meeting another type } 1\right].$$

Since the probability of meeting type 1 is $\rho_1 + (1-\rho_1)(1-p)$, we get

$$u_1^c = \alpha_1[\rho_1 + (1-\rho_1)(1-p)].$$

Therefore the difference between u_0^c and u_1^c is

$$\Delta u^c = \alpha_0[\rho_0 + (1-\rho_0)p] - \alpha_1[\rho_1 + (1-\rho_1)(1-p)]\Big], \qquad \text{[S9a]}$$

which is the difference in utilities due to conformity with their own types. This equation captures how the fraction p of type 1 evolves when conformist benefits are realized *via the pairwise interaction* and the matching is assortative with probabilities ρ_0 and ρ_1.

If $\alpha_0 = \alpha_1 = \alpha$ and $\rho_0 = \rho_1 = \rho$,

$$\Delta u^c = \alpha(1-\rho)(2p-1). \qquad \text{[S9b]}$$

If $\rho = 0$, the model reduces to population-based conformity. In contrast, when $\rho = 1$, all interactions occur exclusively among individuals of the same type, so there is effectively no external pressure to switch types under conformity. Overall, assortative matching reduces the overall force of majority influence, thereby simplifying the conditions under which authorities introduce new strategies.

Bringing all modeling components together, with a single authority

$$\frac{dp}{dt} = p(1-p)[s - 2\sigma\ \bar{\rho}(p) + \Delta u_c]\ \begin{cases} +\beta(1-p) \text{ if worldview } \theta = 0 \text{ is promoted,} \\ -\beta p \text{ if worldview } \theta = 1 \text{ is promoted} \end{cases} \qquad \text{[S10]}$$

Example. In the main text we consider a case when both types have equal conformity parameters ($\alpha_0 = \alpha_1 = \alpha$), type 1 is unbiased ($\delta_1 = 0$) and matches with others randomly ($\rho_1 = 0$) while type 0 is biased ($\delta_1 > 0$) and matches with others assortatively ($\rho_0 > 0$). It is assume that the authority promotes the biased worldview. In this case,

$$s =[q\delta_1 - (1-q)\delta_0](r_0 + r_1) = -(1-q)\delta_0(r_0 + r_1), \qquad \text{[S11a]}$$

$$\sigma =[r_0 q\delta_1 + r_1(1-q)\delta_0] = r_1(1-q)\delta_0, \qquad \text{[S11b]}$$

$$\bar{\rho}(p) =[(1-p)\rho_0 + p\rho_1] = (1-p)\rho_0, \qquad \text{[S11c]}$$

$$\Delta u_c =\alpha[2p - 1 + (1-p)\rho_0]. \qquad \text{[S11d]}$$

Note that $\sigma/|s| = \frac{r_1}{r_0+r_1}$.

Equation (S10) can then be rewritten as

$$\frac{\mathrm{d}p}{\mathrm{d}t} = |s|p(1-p)\left(-1 - 2\frac{\sigma}{|s|}(1-p)\rho_0 + \frac{\alpha}{|s|}[2p - 1 + (1-p)\rho_0]\right) + \frac{\beta}{|s|}(1-p). \qquad \text{[S12]}$$

We show the dependence of the equilibria of this equation on $\alpha/|s|, \beta/|s|$ and ρ_0 in Figure 2 of the main text assuming that $r_0 = r_1$ so that $\sigma/|s| = 1/2$.

Conformity based on observed behavior. The probability that a type $\theta = 0$ agent believes that $e = 0$ is:

$$q \times 1 + (1-q) \times \delta.$$

The probability that a type $\theta = 1$ agent believes that $e = 0$ is:

$$q \times (1-\delta) + (1-q) \times 0.$$

Therefore the frequency of agents who believe that $e = 0$ and behave competitively is

$$P_0 = p[q \times 1 + (1-q) \times \delta] + (1-p)[q \times (1-\delta) + (1-q) \times 0] = q + \delta(p-q).$$

Therefore, with conformity based on observed behavior rather than on worldviews of others, equation (6a) becomes

$$\frac{dp}{dt} = sp(1-p) + \alpha_e p(1-p)[2q - 1 + 2\delta(p-q)].$$

If $\delta \to 0$, conformity will effectively increase the strength of selection s (because s is proportional to $2q-1$. If $q = 1/2$, then the conformity term reduces to $\alpha_e p(1-p) \times \delta(2p-1)$.

Let $\varepsilon = 2q - 1$. Equilibrium $p = 0$ is stable if

$$s + \alpha(1-\delta)\varepsilon < \alpha\delta.$$

Equilibrium $p = 1$ is stable if

$$-s - \alpha(1-\delta)\varepsilon < \alpha\delta.$$

Both equilibria are stable simultaneously and there is an unstable heterogeneous equilibrium separating them if

$$|s + \alpha(1-\delta)\varepsilon| < \alpha\delta.$$

In the simple symmetric model, $s = (r_0 + r_1)\delta e$ (equation 2). In the case the last inequality becomes

$$|e| < \frac{\alpha\delta}{(r_0 + r_1)\delta + \alpha(1-\delta)}.$$
